# Enhanced tumor targeting of cRGD peptide-conjugated albumin nanoparticles in the BxPC-3 cell line

**DOI:** 10.1038/srep31539

**Published:** 2016-08-12

**Authors:** Xinzhe Yu, Yunlong Song, Yang Di, Hang He, Deliang Fu, Chen Jin

**Affiliations:** 1 Pancreatic Surgery Department, Huashan Hospital, Fudan University. Shanghai 200040,China; 2 School of Pharmacy, The Second Military Medical University, Shanghai 200433, P.R.China

## Abstract

The emerging albumin nanoparticle brings new hope for the delivery of antitumor drugs. However, a lack of robust tumor targeting greatly limits its application. In this paper, cyclic arginine-glycine-aspartic-conjugated, gemcitabine-loaded human serum albumin nanoparticles (cRGD-Gem-HSA-NPs) were successfully prepared, characterized, and tested *in vitro* in the BxPC-3 cell line. Initially, 4-*N*-myristoyl-gemcitabine (Gem-C14) was formed by conjugating myristoyl to the 4-amino group of gemcitabine. Then, cRGD-HSA was synthesized using sulfosuccinimidyl-(4-*N*-maleimidomethyl)cyclohexane-1-carboxylate (Sulfo-SMCC) cross-linkers. Finally, cRGD-Gem-HSA-NPs were formulated based on the nanoparticle albumin-bound (nab) technology. The resulting NPs were characterized for particle size, zeta potential, morphology, encapsulation efficiency, and drug loading efficiency. *In vitro* cellular uptake and inhibition studies were conducted to compare Gem-HSA-NPs and cRGD-Gem-HSA-NPs in a human pancreatic cancer cell line (BxPC-3). The cRGD-Gem-HSA-NPs exhibited an average particle size of 160 ± 23 nm. The encapsulation rate and drug loading rate were approximately 83 ± 5.6% and 11 ± 4.2%, respectively. *In vitro*, the cRGD-anchored NPs exhibited a significantly greater affinity for the BxPC-3 cells compared to non-targeted NPs and free drug. The cRGD-Gem-HSA-NPs also showed the strongest inhibitory effect in the BxPC-3 cells among all the analyzed groups. The improved efficacy of cRGD-Gem-HSA-NPs in the BxPC-3 cell line warrants further *in vivo* investigations.

For the past several decades, the 1^st^ line chemotherapy for advanced pancreatic cancer has been gemcitabine-based or fluorouracil-based regimen^1^,^2^. However, neither of these two drugs are satisfactory due to their short plasma half-life and relative low concentration around tumor sites^3^. Furthermore, to achieve optimal clinical efficacy, a frequent administration schedule at a high dose is needed, which leads to significant side effects^4^. Hence, the identification of a novel therapeutic strategy is urgently required. Until recently, albumin nanoparticles (NPs) have been increasingly used as drug carriers based on the following advantages: they are biodegradable in nature, relatively easy to prepare, and often show a controlled release profile^5^,^6^,^7^. Theoretically, nanoparticles can effectively reach tumor sites through two possible mechanisms: i) passive targeting due to the Enhanced Permeability and Retention (EPR) effect^8^ and ii) active targeting based on the attachment of specific ligands to the surface of nanoparticles, which enables nanoparticles binding to receptors that are overexpressed by cancer cells or angiogenic endothelial cells^9^. EPR and the overexpression of angiogenesis-related surface receptors are the key features of tumor blood vessels. Hence, combination of EPR-mediated passive targeting and specific ligand-mediated active targeting has received notable attention in the past years^10^.

Integrins, which consist of an α and a β subunit, are a family of cell surface receptors that are responsible for anchoring cells to the extracellular matrix^11^. Recently, they were shown to mediate and diversify biological communication involving cell adhesion and signal transduction^12^,^13^. Among the various subtypes, α_V_β3 integrins are the most promising therapeutic target because they are overexpressed on the actively proliferating endothelium of tumor tissues and are determinants on angiogenic endothelium^14^,^15^,^16^. It is generally believed that α_V_β3 integrins specifically recognize the RGD motif^17^,^18^, which suggests the possibility of using RGD-conjugated carriers to deliver drugs into cancer cells as active tumor targeting therapy^19^,^20^,^21^. Although RGD peptides conjugated drug delivery systems aiming at α_v_β3 integrin for active tumor targeting therapy have been well documented in several platforms such as liposome^22^, copolymer^23^, iron oxide magnetic nanoparticles^24^, rare studies are focusing on the albumin platform. Integrin α_v_β3 is expressed in approximately 58% of human pancreatic tumors and is associated with increased lymph node metastasis^18^. In preliminary experiments, we screened several human pancreatic cancer cell lines and chose BxPC-3, which has high α_v_β3 integrin expression, as the targeting cell line for our *in vitro* studies^25^.

We previously prepared gemcitabine-loaded albumin nanoparticles using the nab technology^26^. Our *in vivo* results confirmed that the prepared Gem-HSA-NPs effectively inhibited the growth of pancreatic cancer cell lines with moderate toxicity. In this study, we aimed to further enhance tumor targeting by conjugating the cRGD peptide to the surface of the Gem-HSA-NPs. To the best of our knowledge, this is the first report on using the nab technology to prepare the cRGD-Gem-HSA-NPs. Here, we describe the synthesis and characterization of the cRGD-Gem-HSA-NPs. Furthermore, the targeting efficiency of the cRGD peptide was qualitatively and quantitatively evaluated *in vitro* at the cellular level. Finally, the cRGD-Gem-HSA-NPs were evaluated for *in vitro* biological inhibition activity in the BxPC-3 cell line.

## Materials and Methods

### Chemical and reagents

Gemcitabine was got from Eli Lilly and Company (Indiana, USA). HSA (20%, 50 mL) was purchased from the Baxter AG (Vienna, Austria). cRGD (sequence: Arg-Gly-Asp-D-Tyr-Lys) was synthesized by Shanghai Gil Biochemistry Co., Ltd. (Shanghai, China); the purity was greater than 99.9%. Sulfo-SMCC (sulfosuccinimidyl-(4-*N*-maleimidomethyl)cyclohexane-1-carboxylate) was obtained from Thermo Fisher Scientific (Waltham, MA, USA). Normal saline (NS) was purchased from Shanghai Baxter Healthcare Co., Ltd. (Shanghai, China). Absolute ethanol and chloroform were purchased from Sinopharm Chemical Reagent Co., Ltd. (Shanghai, China). Deionized water was purchased from Fudan University (Shanghai, China). Roswell Park Memorial Institute (RPMI)-1640 culture medium and fetal bovine serum (FBS) were obtained from Gibco (Carlsbad, CA, USA). Double stain Hoechst/PI kit and myristic acid (C-14) were purchased from Sigma-Aldrich (St. Louis, MO, USA). Cell Counting Kit-8 (CCK-8) was purchased from Dojindo (Kyushu, Japan). PBS and other reagents were prepared in our laboratory. All the solvents and chemicals were of analytical grade.

### Synthesis of 4-*N*-myristoyl-gemcitabine (Gem-C14)

**Figure 1:**
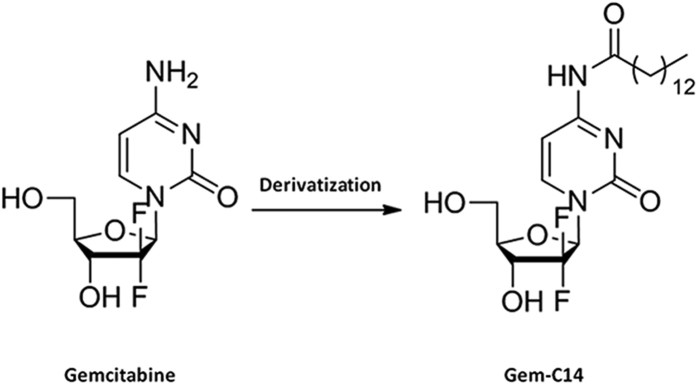
A schematic illustration of the synthesis of 4-*N*-myristoyl-gemcitabine (Gem-C14).

The synthesis of Gem-C14 was described in details elsewhere^27^. In short, myristic acid and 1,1′-carbonyldiimidazole (CDI) was mixed and stirred in tetrahydrofuran, which was then added into the CH_3_CN solution of gemcitabine base, pyridine and trimethylsilyl chloride. The resulting mixture was stirred overnight at 60 °C. Trifluoroacetic acid was added dropwise into the solution. The reaction mixture was stirred at room temperature and then concentrated under reduced pressure. The crude was purified by column chromatography and further recrystallized to provide the final product. A schematic illustration of the synthesis of Gem-C1was shown in Fig. 1.

### The synthesis of cRGD-HSA

#### Activation of HSA with Sulfo-SMCC

To activate HSA, Sulfo-SMCC was dissolved in distilled water by gentle agitation with mild heat (~40 °C) and diluted to 2.5 mg/mL with PBS. A 20 molar excess of Sulfo-SMCC was added to HSA in 15 mL of PBS at *p*H 7.8 for a final HSA concentration of 2 mg/mL. The reaction was allowed to proceed under constant agitation for 5 h under a nitrogen atmosphere. The mixture was dialyzed in PBS buffer at 4 °C and then freeze-dried with a lyophilizer.

#### Conjugation of sulfhydryl-containing cRGD to maleimide-activated HSA

**Figure 2:**
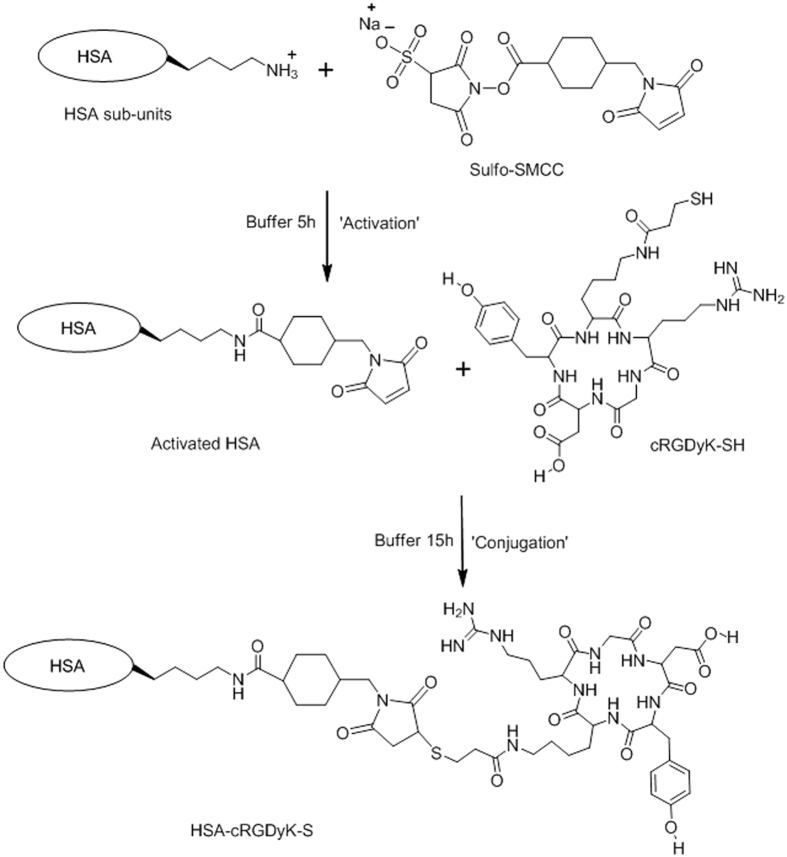
A schematic illustration of the synthesis of cRGD-HSA.

The molar ratio of maleimide-activated HSA to cRGD in the final conjugation reaction was 1:15. Briefly, the sulfhydryl-containing RGD and the activated HSA were dissolved in PBS buffer; then, the sulfhydryl-containing cRGD solution was slowly added to the activated HSA PBS buffer. The reaction was allowed to proceed at room temperature for 15 h. To avoid unwanted oxidation of sulfhydryl groups, all the reactions and procedures were performed under argon gas and with argon-bubbled PBS buffer. A schematic illustration of the synthesis of cRGD-HSA was shown in Fig. 2. All the products were analyzed by 1H-NMR and Matrix-Assisted Laser Desorption/Ionization Time of Flight Mass Spectrometry (MALDI-TOF-MS).

### Preparation and Characterization of cRGD-Gem-HSA-NPs

The cRGD-Gem-HSA-NPs were prepared by the nab technology as previously reported^28^. Briefly, cRGD-HSA was mixed with pure water. Separately, Gem-C14 was dissolved in chloroform saturated with pure water. These two solutions were then fully mixed and homogenized (Nano DeBEE manufactured by BEE International, MA, USA) at 20,000 psi for nine cycles. The generated colloid was rotary evaporated to remove chloroform at 25 °C for 15 min under vacuum. The obtained nanoparticles were then filtered through a 0.25-μm membrane syringe filter, and the solvent was removed by lyophilization for 48 h at −80 °C. The obtained cRGD-Gem-HSA-NP powder was vacuum-dried for 48 h and stored at room temperature.

The preparation of coumarin-6 (C6)-labeled HSA-NPs and C6-cRGD-HSA-NPs followed the same procedure as that for cRGD-Gem-HSA-NPs. Finally, the nanoparticles were run over a sepharose CL-4B column to remove the free coumarin-6.

The particle size and zeta potential were acquired using Zetasizer (Malvern ZS, UK) at a scattering angle of 120°. The nanoparticle suspension was added dropwise onto the copper grids, which were then dried at room temperature. Transmission electron microscopy (TEM) images of the nanoparticles were acquired under 20,000X and 50,000X magnification respectively (Hitachi H-600, Japan).

To determine the drug loading efficiency and encapsulation efficiency, a pre-weighed aliquot of cRGD-Gem-HSA-NPs was placed in distilled water and sonicated to extract the Gem-C14. The extracted Gem-C14 was analyzed using a high-performance liquid chromatography (HPLC)-UV assay. Data were collected, and the efficiencies were calculated using the following formulas:


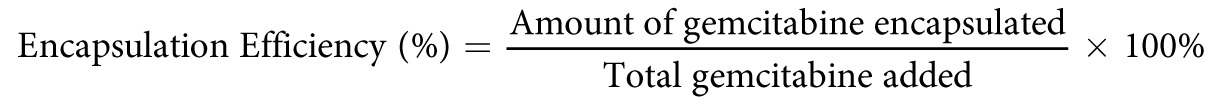



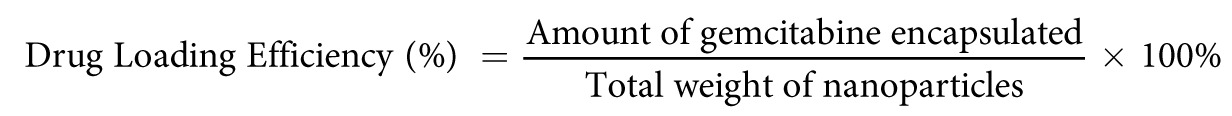


### Cell culture

The BxPC-3 cell line (human pancreatic cancer, originally from American Type Culture Collection) was purchased from the Shanghai Branch of the Chinese Academy of Sciences (Shanghai, China).

Cells were cultured at 37 °C in the presence of 5% CO_2_ and 95% air with >95% humidity. Cells were grown in RPMI-1640 containing 10% FBS, 100 U/mL penicillin and 100 mg/mL streptomycin.

### Qualitative confocal microscopy analysis

The BxPC-3 cells were seeded in 6-well plates at a density of 1 × 10^4^ cells per well. After incubation with coumarin-6 (C6)-labeled HSA-NPs and C6-cRGD-HSA-NPs, cells were immediately washed with PBS and fixed in paraformaldehyde for 10 min, followed by cell nuclei staining with DAPI for 10 min. Cover slips were mounted on slides after three washes with PBS, and the slides were analyzed by confocal microscopy (Leica DMI 4000B, Germany).

### Quantitative flow cytometry analysis

The BxPC-3 cells were seeded at a density of 1 × 10^5^ cells/well in 6-well plates. After incubation with C6-HSA-NPs and C6-cRGD-HSA-NPs, the cells were washed three times with PBS, trypsinized and centrifuged at 2000 rpm for 4 min to obtain a cell pellet, which was subsequently resuspended in PBS and analyzed by flow cytometry (BD Biosciences). For each sample, 10,000 events were collected, and data were analyzed with Cell-Quest software.

### Cell viability

The *in vitro* cytotoxic effects of gemcitabine, Gem-C14, Gem-HSA-NPs, and cRGD-Gem-HSA-NPs in the BxPC-3 cells were determined using the CCK-8 assay. Briefly, cells were seeded into 96-well culture plates (10,000 cells/well) and incubated overnight at 37 °C, 5% CO_2_. Then, the cells were incubated with various concentrations of gemcitabine, Gem-C14, Gem-HSA-NPs, and cRGD-Gem-HSA-NPs for 48 h. Cell viability was quantitated based on the dye absorption at 450 nm, which was determined using an automatic multiwell spectrophotometer. The cell inhibition rate (%) was calculated as follows: [1 −(absorbance of the study group/absorbance of the control group)]*100.

### Apoptotic cell damage assays

The apoptosis rate was assessed using Annexin V-FITC/PI according to the manufacturer’s instructions. The BxPC-3 cells were grouped and treated as described for the CCK-8 assay. Briefly, the BxPC-3 cells were placed in a 6-well culture plate (5 × 10^5^ cells/well). The cells in each group were collected through centrifugation at 1000 rpm for 5 min, washed three times with PBS and resuspended in binding buffer. Then, 5 μL of Annexin V-FITC solution and 10 μl of PI solution were added. The stained cells were incubated for 15 min at room temperature in the dark. Finally, the suspension was subjected to FCM analysis (BD Biosciences). All the experiments were performed in triplicate.

### Statistical analysis

All the data are presented as the mean ± standard deviation (SD). Significant differences between two groups were determined using Student’s t-test, while multiple groups were analyzed by one-way ANOVA with Fisher’s LSD. All the statistical analyses were performed with SPSS software (version 22.0; IBM Inc., New York, NY, USA). A value of *p* < 0.05 was considered significant.

## Results and Discussion

### Synthesis of 4-*N*-myristoyl-gemcitabine (Gem-C14)

In order to obtain a gemcitabine prodrug with satisfactory lipophilicity for albumin encapsulation, we designed and synthesized 4-*N*-myristoyl-(C14) gemcitabine derivative named Gem-C14. After analyzed by^1^H-NMR, Gem-C14 had a purity greater than 99.9%. 1H NMR (DMSO-d6, 400 MHz) δ:11.0 (1H, s), 8.25 (1H, d, J = 7.5 Hz), 7.29 (1H, d, J = 7.5 Hz), 6.34 (1H, d, J = 6.6 Hz), 6.17 (1H, t, J = 7.5 Hz), 5.32 (1H, brs), 4.20 (1H, m), 3.88 (1H, m), 3.83 (1H, m), 3.65 (1H, m), 2.39 (2H, t, J = 7.2 Hz), 1.54 (2H, m), 1.24 (20H, brs), 0.85 (3H, t, J = 7.2 Hz).

### Characterization of the cRGD-Gem-HSA-NPs

**Figure 3:**
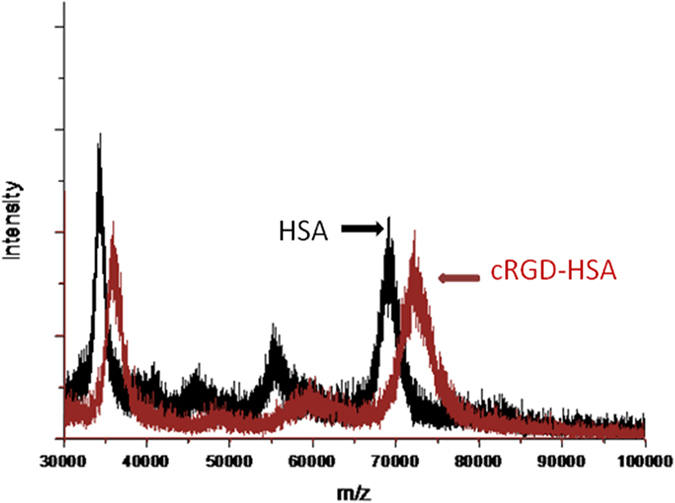
cRGD-HSA and HSA was analyzed by Matrix-Assisted Laser Desorption/Ionization Time of Flight Mass Spectrometry respectively. The mass-to-charge ratio (m/z) of the red peak (cRGD-HSA) was approximately 3000 higher than that of the black peak (HSA), which demonstrated several cRGD were successfully conjugated to the surface of HSA.

**Figure 4:**
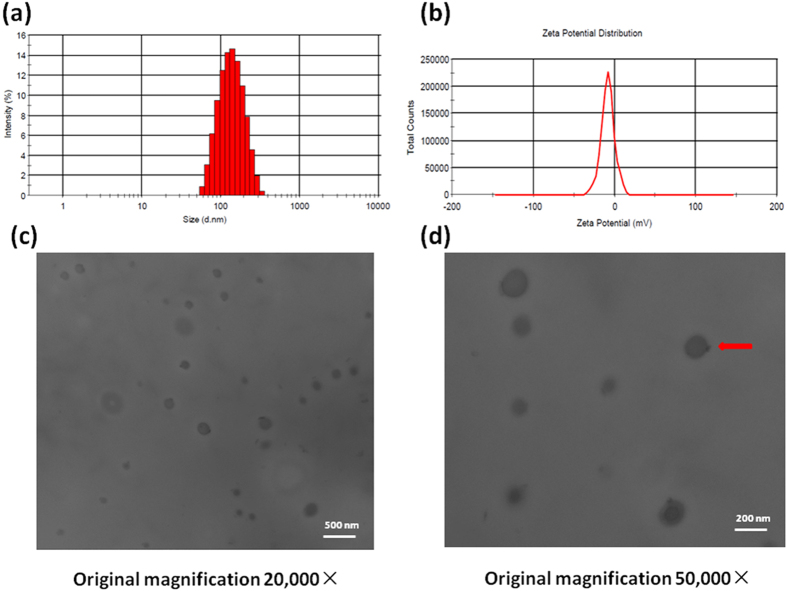
Particle size and size distribution of cRGD-Gem-HSA-NPs determined using (**a**) Malvern Zetasizer (**a**). Zeta potential spectrum of cRGD-Gem-HSA-NPs in PBS solution (**b**). TEM image of cRGD-Gem-HSA-NPs under 20,000X magnification (**c**). TEM image of cRGD-Gem-HSA-NPs under 50,000X magnification. Inset: a red arrow indicating the morphology of the cRGD-Gem-HSA-NPs (**d**).

The cRGD-Gem-HSA-NPs were formulated based on the nab technology, in which Gem-C14 was mixed with cRGD-HSA in an aqueous solvent and passed under high pressure through a jet to form drug albumin nanoparticles. To validate the cRGD conjugation, we used MALDI-TOF-MS to compare the average molecular weight change between HSA and cRGD-HSA. As indicated in Fig. 3, the mass-to-charge ratio (m/z) of the red peak (cRGD-HSA) was approximately 3000 higher than that of the black peak (HSA). Because the molecular weights of cRGD and Sulfo-SMCC are 620 and 436, respectively, we assumed that at least three cRGD molecules were successfully conjugated to the surface of each HSA molecule. The size and surface charge of the complex were measured at various weight ratios. As shown in Fig. 4a, the average size of the nanoparticles was 160 ± 23 nm, and the PDI was 0.123 ± 0.01. Overall, the nanoparticles showed a narrow size distribution and good water solubility, suggesting their potential utility as an active targeting anti-tumor agent^29^,^30^,^31^. The nanoparticles showed a negative surface potential of −8.26 ± 2.1 mV, (Fig. 4b) indicating a good dispersity and stability in human blood, the latter may be conductive to a prolonged blood circulation; The morphology of the cRGD-Gem-HSA-NPs was observed by TEM. Under 20,000X magnification, the cRGD-Gem-HSA-NPs exhibited a nearly spherical shape with a moderately uniform particle size and an even distribution (Fig. 4c). Under 50,000X magnification, the nanoparticles had a brighter core surrounded by a dark membrane, which confirmed the distinct layer (Fig. 4d).

The encapsulation efficiency of cRGD-Gem-HSA-NPs was 83 ± 5.6%, while the drug loading efficiency was 11 ± 4.2%; both values corresponded with our optimal expectations. We used an RGD pre-modification method to prepare the cRGD-Gem-HSA-NPs: we initially synthesized cRGD-HSA using the linker Sulfo-SMCC and then encapsulated Gem-C14 into cRGD-Gem-HSA-NPs. Rather than the conjugation of cRGD to the surface of Gem-HSA-NPs, the latter may cause drug loss and leakage during the synthesis and purification process.

### Cellular uptake of coumarin-6-labeled nanoparticles

**Figure 5:**
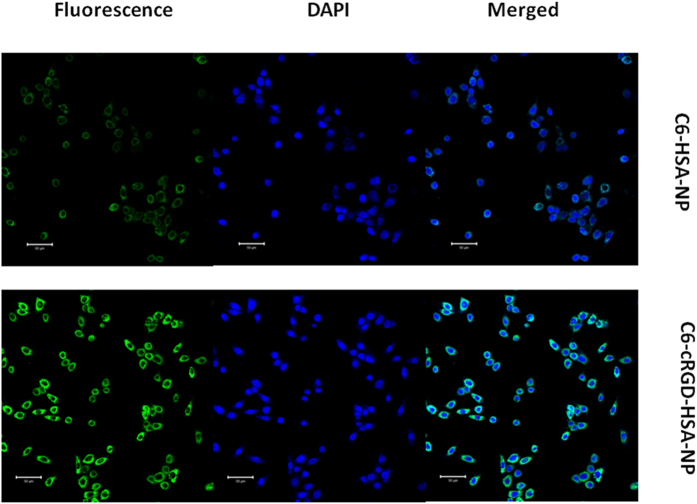
Confocal images of the BxPC-3 cells incubated with coumarin-6 (C6)-labeled HSA-NPs and coumarin-6-labeled cRGD-HSA-NPs. Green: fluorescence of C6-HSA-NPs and C6-cRGD-HSA-NPs. Blue: DAPI (nucleus). Merged: fluorescence (green) and DAPI (blue). Bar: 50 μm.

Integrin α_V_β3 is the predominant receptor of the RGD motif and is highly expressed in tumor cells and neo-vascular endothelial cells. The application of RGD peptides as coating surface to enhance drug delivery has been broadly investigated. In this study, we chose cyclic RGD peptides rather than linear RGD peptides due to their higher affinity for α_V_β3 integrins^32^,^33^. Coumarin-6-labeled nanoparticles were used to investigate the cellular uptake characteristics of cRGD^34^ the qualitative and quantitative results were obtained by fluorescent imaging and flow cytometry, respectively. As shown in Fig. 5, the BxPC-3 cells treated with cRGD-C6-HSA-NPs exhibited significantly higher green fluorescence intensity than those treated with C6-HSA-NPs.

**Figure 6:**
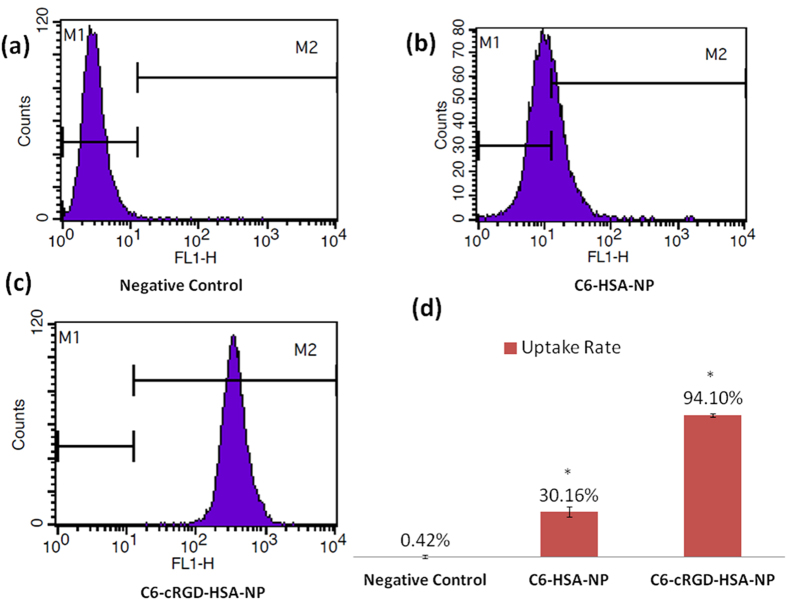
Typical flow cytometry plots of the BxPC-3 cells after (**a**) 4-h incubation with sterile PBS (**a**), C6-HSA-NPs (**b**), or C6-cRGD-HSA-NPs (**c**). The proportion of the purple peak that falls within the M2 range represents the captured C6-positive cells. The lower right column graph shows the mean fluorescence intensity of the cells in the three groups. Data are presented as the mean ± SD (n = 3). These results confirmed that decorating the particle surface with cRGD significantly facilitated the uptake of nanoparticles in the BxPC-3 cells. *p < 0.01, compared with the negative control.

In the quantitative analysis of the cellular uptake of the cRGD peptide, cRGD-C6-HSA-NPs showed the highest cellular uptake into the BxPC-3 cells (94.10 ± 1.2%, Fig. 6c); non-targeting cRGD-conjugated nanoparticles, C6-HSA-NPs, showed a much lower cellular uptake (30.16 ± 3.5%; *p* < 0.05, Fig. 6b); while the negative control group showed the lowest cellular uptake (0.42 ± 0.08%, Fig. 6a). After conjugation to the cRGD motif, the cellular uptake activity of cRGD-C6-HSA-NPs increased 3-fold compared with that of C6-HSA-NPs (Fig. 6). These results confirmed that decorating the particle surface with cRGD significantly facilitated the uptake of nanoparticles in the BxPC-3 cells (Fig. 6d).

### *In vitro* inhibition and apoptosis

**Figure 7:**
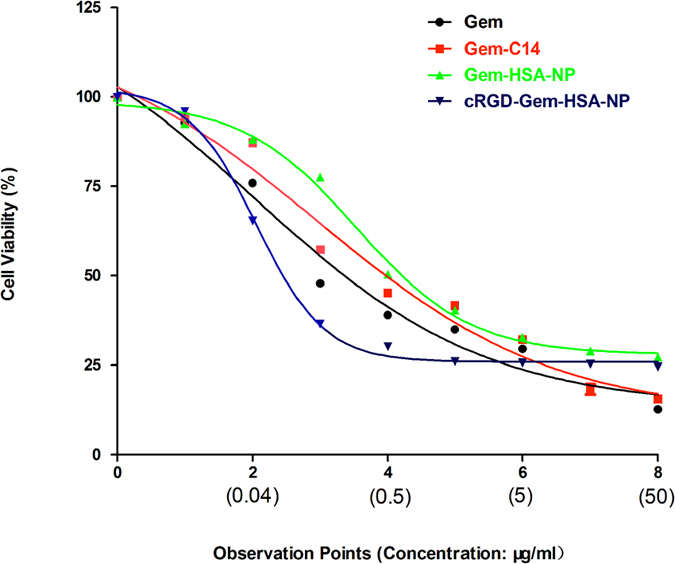
*In vitro* cytotoxicity of cRGD-Gem-HSA-NP in the BxPC-3 cell line. Experimental points 1–8 correspond to doses of 0.01, 0.04, 0.2, 0.5, 1, 5, 10, and 50 μg/mL, respectively. All formulations inhibited cell growth in a dose-dependent manner; cRGD-Gem-HSA-NP exhibited the highest cytotoxicity, followed sequentially by gemcitabine, Gem-C14, and Gem-HSA-NP. The IC50 was 0.1 μg/mL for cRGD-Gem-HSA-NP, 0.28 μg/mL for gemcitabine, 0.38 μg/mL for Gem-C14, and 0.42 μg/mL for Gem-HSA-NP. For ease of comparison, all the IC50 values shown here were calculated on the basis of the amount of the equivalent gemcitabine base.

The *in vitro* cytotoxicity of various Gem formulations, including Gem, Gem-C14, Gem-HSA-NPs, and cRGD-Gem-HSA-NPs, was evaluated in the human pancreatic cancer cell line via the CCK-8 assay. In Fig. 7, experimental points 1–8 correspond to doses of 0.01, 0.04, 0.2, 0.5, 1, 5, 10, and 50 μg/mL, respectively. All four Gem formulations inhibited cell growth in a dose-dependent manner. At various concentrations, cRGD-Gem-HSA-NPs exhibited the strongest cytotoxicity, followed sequentially by Gem, Gem-C14, and Gem-HSA-NPs. The IC50 was 0.10 μg/mL for cRGD-Gem-HSA-NP, 0.28 μg/mL for Gem, 0.38 μg/mL for Gem-C14, and 0.42 μg/mL for Gem-HSA-NP. Notably, this result was consistent with the cellular uptake study. The Gem-HSA-NPs showed the least cytotoxicity; however, after cRGD conjugation, the tumor targeting ability of the nanoparticles was strongly enhanced.

**Figure 8:**
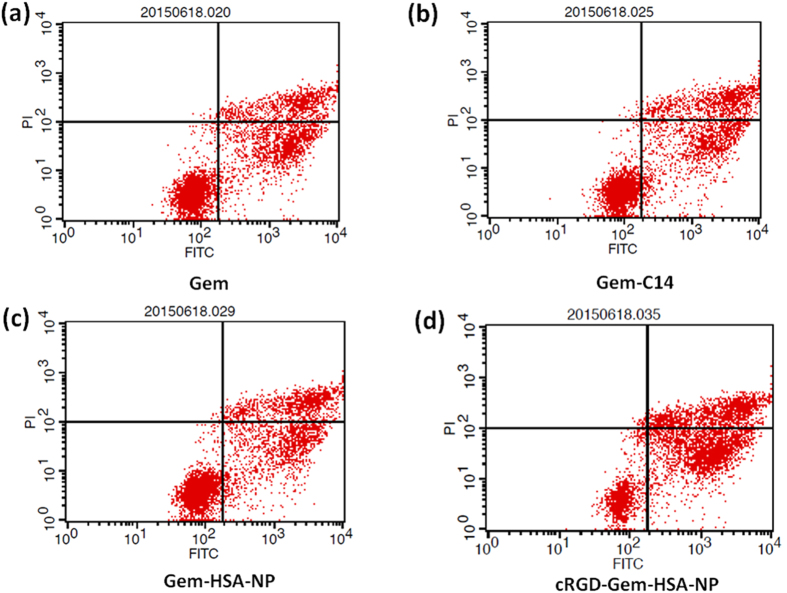
Annexin V-FITC/PI flow cytometry plots for gemcitabine (**a**), Gem-14 (**b**), Gem-HSA-NP (**c**), and cRGD-Gem-HSA-NP (**d**). The apoptotic rates of the four drug formulations were 34.94 ± 2.5%, 37.72 ± 1.8%, 37.48 ± 2.6%, and 56 ± 2.1%, respectively. Compared with the first three groups, the cRGD-Gem-HSA-NP group showed a significantly higher apoptosis rate (*p* < 0.05). Numbers in the quadrants represent the percentage of cells in early apoptosis (UR), of cells in late apoptosis or necrosis (LR), and of cell debris (UL). Abbreviations: FITC, fluorescein isothiocyanate.

We further assessed the apoptosis assay of cells treated with the four formulations. As shown in Fig. 8, apoptosis and necrosis occurred in all treatment groups and the total apoptosis rates (including early and late stage apoptosis) of the Gem (a), Gem-C14 (b), Gem-HSA-NP (c), and cRGD-Gem-HSA-NP groups (d) were 34.94 ± 2.5%, 37.72 ± 1.8%, 37.48 ± 2.6%, and 56 ± 2.1%, respectively. Compared with the first three groups, the cRGD-Gem-HSA-NP group showed a significantly higher apoptosis rate (*p* < 0.05). This result was consistent with the cytotoxicity study, and it indicated that cRGD-Gem-HSA-NPs more strongly induced early and late apoptosis due to greater Gem intracellular uptake because of the active targeting by the cRGD peptide, which produced higher cytotoxicity than the other formulations.

One limitation of this study is that all our therapeutic studies were at the *in vitro* level. Pancreatic cancer is characterized for its abundant stroma, the latter may lead to a chemical and physical drug barrier. The two advantages of our cRGD-Gem-HSA-NPs are active tumor targeting and passive tumor targeting by virtue of EPR effect. *In vivo* study will leverage this feature into maximum therapy efficacy. Therefore, more *in vivo* tests should be further verified.

Gemcitabine, acting as a cancer-chemotherapeutic agent, has no specificity to pancreatic cancer cells and frequently displays toxicity. In this research, on the one hand, we encapsulated gemcitabine into nanoparticles to achieve passive tumor targeting by virtue of EPR effect; on the other hand, we further decorated the surface of nanoparticles with cRGD to attain active tumor targeting. As far as we know, this is the first study report on the use of the nab technology to prepare the cRGD-Gem-HSA-NPs, which produced significantly improved antitumor efficacy compared with the control groups. The enhanced *in vitro* efficacy of cRGD-Gem-HSA-NPs towards a pancreatic cancer cell line paving the way for further *in vivo* studies.

## Conclusion

In summary, we have successfully prepared cRGD-Gem-HSA-NPs. The *in vitro* results confirmed that cRGD-anchored nanoparticles can deliver gemcitabine to a pancreatic cancer cell line more efficiently. Additional *in vivo* studies are warranted to establish the therapeutic potential of cRGD-Gem-HSA-NPs in combating pancreatic cancer. Furthermore, the cRGD-conjugated albumin nanoparticle platform in this study paves a new way for smart delivery of other anticancer drugs to human cancers.

## Additional Information

**How to cite this article**: Yu, X. *et al*. Enhanced tumor targeting of cRGD peptide-conjugated albumin nanoparticles in the BxPC-3 cell line. *Sci. Rep.* **6**, 31539; doi: 10.1038/srep31539 (2016).

## References

[b1] Hidalgo M. Pancreatic cancer. N Engl J Med 362, 1605–1617, doi: 1 0.1056/NEJMra0901557 (2010). 20427809 10.1056/NEJMra0901557

[b2] King R. S. Gemcitabine. New first-line therapy for pancreatic cancer. Cancer Pract 4, 353–354 (1996). 9128490

[b3] Wang L. . GEM-loaded magnetic albumin nanospheres modified with cetuximab for simultaneous targeting, magnetic resonance imaging, and double-targeted thermochemotherapy of pancreatic cancer cells. Int J Nanomedicine 10, 2507–2519, doi: 10.2147/IJN.S77642 (2015). 25848268 PMC4386779

[b4] Morris S. L., Beasley M. & Leslie M. Chemotherapy for pancreatic cancer. N Engl J Med 350, 2713–2715; author reply 2713-2715, doi: 10.1056/NEJM200406243502617 (2004). 15215490

[b5] Von Hoff D. D. . Increased survival in pancreatic cancer with nab-paclitaxel plus gemcitabine. N Engl J Med 369, 1691–1703, doi: 10.1056/NEJMoa1304369 (2013). 24131140 PMC4631139

[b6] Ming X., Carver K. & Wu L. Albumin-based nanoconjugates for targeted delivery of therapeutic oligonucleotides. Biomaterials 34, 7939–7949, doi: 10.1016/j.biomaterials.2013.06.066 (2013). 23876758 PMC3810404

[b7] Elzoghby A. O., Samy W. M. & Elgindy N. A. Albumin-based nanoparticles as potential controlled release drug delivery systems. J Control Release 157, 168–182, doi: 10.1016/j.jconrel.2011.07.031 (2012). 21839127

[b8] Maeda H., Wu J., Sawa T., Matsumura Y. & Hori K. Tumor vascular permeability and the EPR effect in macromolecular therapeutics: a review. J Control Release 65, 271–284 (2000). 10699287 10.1016/s0168-3659(99)00248-5

[b9] Danhier F., Feron O. & Preat V. To exploit the tumor microenvironment: Passive and active tumor targeting of nanocarriers for anti-cancer drug delivery. J Control Release 148, 135–146, doi: 10.1016/j.jconrel.2010.08.027 (2010). 20797419

[b10] Kunjachan S. . Passive versus active tumor targeting using RGD- and NGR-modified polymeric nanomedicines. Nano Lett 14, 972–981, doi: 10.1021/nl404391r (2014). 24422585 PMC3940962

[b11] Ivanov B., Grzesik W. & Robey F. A. Synthesis and use of a new bromoacetyl-derivatized heterotrifunctional amino acid for conjugation of cyclic RGD-containing peptides derived from human bone sialoprotein. Bioconjug Chem 6, 269–277 (1995). 7632798 10.1021/bc00033a006

[b12] Coopman P. J., Thomas D. M., Gehlsen K. R. & Mueller S. C. Integrin alpha 3 beta 1 participates in the phagocytosis of extracellular matrix molecules by human breast cancer cells. Mol Biol Cell 7, 1789–1804 (1996). 8930900 10.1091/mbc.7.11.1789 PMC276026

[b13] Hynes R. O. Integrins: versatility, modulation, and signaling in cell adhesion. Cell 69, 11–25 (1992). 1555235 10.1016/0092-8674(92)90115-s

[b14] Jin H. & Varner J. Integrins: roles in cancer development and as treatment targets. Br J Cancer 90, 561–565, doi: 10.1038/sj.bjc.6601576 (2004). 14760364 PMC2410157

[b15] Dubey P. K., Singodia D., Verma R. K. & Vyas S. P. RGD modified albumin nanospheres for tumour vasculature targeting. J Pharm Pharmacol 63, 33–40, doi: 10.1111/j.2042-7158.2010.01180.x (2011). 21155813

[b16] Hynes R. O. A reevaluation of integrins as regulators of angiogenesis. Nat Med 8, 918–921, doi: 10.1038/nm0902-918 (2002). 12205444

[b17] Zheng Y. . FITC-conjugated cyclic RGD peptides as fluorescent probes for staining integrin alphavbeta3/alphavbeta5 in tumor tissues. Bioconjug Chem 25, 1925–1941, doi: 10.1021/bc500452y (2014). 25312799 PMC4240344

[b18] Hosotani R. . Expression of integrin alphaVbeta3 in pancreatic carcinoma: relation to MMP-2 activation and lymph node metastasis. Pancreas 25, e30–35 (2002). 12142752 10.1097/00006676-200208000-00021

[b19] Zhong Y. . cRGD-directed, NIR-responsive and robust AuNR/PEG-PCL hybrid nanoparticles for targeted chemotherapy of glioblastoma *in vivo*. J Control Release 195, 63–71, doi: 10.1016/j.jconrel.2014.07.054 (2014). 25108151

[b20] Wang W. . RGD Peptides-Conjugated Pluronic Triblock Copolymers Encapsulated with AP-2alpha Expression Plasmid for Targeting Gastric Cancer Therapy *in vitro* and *in vivo*. Int J Mol Sci 16, 16263–16274, doi: 10.3390/ijms160716263 (2015). 26193262 PMC4519948

[b21] Ge Z. . Targeted gene delivery by polyplex micelles with crowded PEG palisade and cRGD moiety for systemic treatment of pancreatic tumors. Biomaterials 35, 3416–3426, doi: 10.1016/j.biomaterials.2013.12.086 (2014). 24439417

[b22] Wang F., Chen L., Zhang R., Chen Z. & Zhu L. RGD peptide conjugated liposomal drug delivery system for enhance therapeutic efficacy in treating bone metastasis from prostate cancer. J Control Release 196, 222–233, doi: 10.1016/j.jconrel.2014.10.012 (2014). 25456829

[b23] Xu Q. . Anti-tumor activity of paclitaxel through dual-targeting carrier of cyclic RGD and transferrin conjugated hyperbranched copolymer nanoparticles. Biomaterials 33, 1627–1639, doi: 10.1016/j.biomaterials.2011.11.012 (2012). 22118775

[b24] Zheng S. W. . RGD-conjugated iron oxide magnetic nanoparticles for magnetic resonance imaging contrast enhancement and hyperthermia. J Biomater Appl 28, 1051–1059, doi: 10.1177/0885328213493486 (2014). 23796630

[b25] Ji S. . RGD-conjugated albumin nanoparticles as a novel delivery vehicle in pancreatic cancer therapy. Cancer Biol Ther 13, 206–215, doi: 10.4161/cbt.13.4.18692 (2012). 22354009

[b26] Yu X. . An *in vitro* and *in vivo* study of gemcitabine-loaded albumin nanoparticles in a pancreatic cancer cell line. Int J Nanomedicine 10, 6825–6834, doi: 10.2147/IJN.S93835 (2015). 26586944 PMC4636168

[b27] Immordino M. L. . Preparation, characterization, cytotoxicity and pharmacokinetics of liposomes containing lipophilic gemcitabine prodrugs. J Control Release 100, 331–346, doi: 10.1016/j.jconrel.2004.09.001 (2004). 15567500

[b28] Kim T. H. . Preparation and characterization of water-soluble albumin-bound curcumin nanoparticles with improved antitumor activity. Int J Pharm 403, 285–291, doi: 10.1016/j.ijpharm.2010.10.041 (2011). 21035530

[b29] Wang S. . Magnetic graphene-based nanotheranostic agent for dual-modality mapping guided photothermal therapy in regional lymph nodal metastasis of pancreatic cancer. Biomaterials 35, 9473–9483, doi: 10.1016/j.biomaterials.2014.07.064 (2014). 25175596

[b30] Jiang X. . Integrin-facilitated transcytosis for enhanced penetration of advanced gliomas by poly(trimethylene carbonate)-based nanoparticles encapsulating paclitaxel. Biomaterials 34, 2969–2979, doi: 10.1016/j.biomaterials.2012.12.049 (2013). 23380351

[b31] Jiang X. . Solid tumor penetration by integrin-mediated pegylated poly(trimethylene carbonate) nanoparticles loaded with paclitaxel. Biomaterials 34, 1739–1746, doi: 10.1016/j.biomaterials.2012.11.016 (2013). 23211449

[b32] Juan H. F. . Proteomics analysis of a novel compound: cyclic RGD in breast carcinoma cell line MCF-7. Proteomics 6, 2991–3000, doi: 10.1002/pmic.200500435 (2006). 16619296

[b33] Kim H. A., Nam K. & Kim S. W. Tumor targeting RGD conjugated bio-reducible polymer for VEGF siRNA expressing plasmid delivery. Biomaterials 35, 7543–7552, doi: 10.1016/j.biomaterials.2014.05.021 (2014). 24894645 PMC4090046

[b34] Jiang X. . Self-aggregated pegylated poly (trimethylene carbonate) nanoparticles decorated with c(RGDyK) peptide for targeted paclitaxel delivery to integrin-rich tumors. Biomaterials 32, 9457–9469, doi: 10.1016/j.biomaterials.2011.08.055 (2011). 21911250

